# Glucose Sensor Using U-Shaped Optical Fiber Probe with Gold Nanoparticles and Glucose Oxidase

**DOI:** 10.3390/s18041217

**Published:** 2018-04-16

**Authors:** Kuan-Chieh Chen, Yu-Le Li, Chao-Wei Wu, Chia-Chin Chiang

**Affiliations:** 1 Department of Mechanical Engineering, National Kaohsiung University of Science and Technology, No. 415, Jiangong Rd., Sanmin Dist., Kaohsiung City 807, Taiwan; 1102403105@gm.kuas.edu.tw (K.-C.C.); 1105303102@gm.kuas.edu.tw (Y.-L.L.); 2 Department of Aeronautical and Mechanical Engineering, Air Force Academy, Academy, No. Sisou 1, Jieshou W. Road, Kaohsiung 820, Taiwan; 1102403104@gm.kuas.edu.tw

**Keywords:** glucose sensor, glucose oxidase, gold nanoparticles, U-shape optical fiber

## Abstract

In this study, we proposed a U-shaped optical fiber probe fabricated using a flame heating method. The probe was packaged in glass tube to reduce human factors during experimental testing of the probe as a glucose sensor. The U-shaped fiber probe was found to have high sensitivity in detecting the very small molecule. When the sensor was dipped in solutions with different refractive indexes, its wavelength or transmission loss changed. We used electrostatic self-assembly to bond gold nanoparticles and glucose oxidase (GOD) onto the sensor’s surface. The results over five cycles of the experiment showed that, as the glucose concentration increased, the refractive index of the sensor decreased and its spectrum wavelength shifted. The best wavelength sensitivity was 2.899 nm/%, and the linearity was 0.9771. The best transmission loss sensitivity was 5.101 dB/%, and the linearity was 0.9734. Therefore, the proposed U-shaped optical fiber probe with gold nanoparticles and GOD has good potential for use as a blood sugar sensor in the future.

## 1. Introduction

Upon its initial development, optical fiber was first used in communications and data transfer applications. Silicon and polymer optical fiber communication technology was further improved upon over the following decades and was also utilized in a variety of other applications due its numerous advantages, including its small size, high sensitivity, imperviousness to electromagnetic waves, and instant responsiveness to other environmental phenomena, among others. It has been widely used, for example, in temperature sensing, stress sensing [1], liquid level monitoring [2], and biomedicine applications. Moreover, because optical fiber sensors can be used for a wide range of applications, they have been put to various uses by the defense, civil construction, and aerospace industries, among other important industries.

Several studies investigated the use of optical fiber sensors for glucose sensing. In 2008, Kwon et al. [3] presented two different types of micro-ring (MRR) shock glucose sensors that they used to measure different temperatures of glucose solutions with concentrations of 0–3%. The sensor sensitivity concentration of MRR1 (the first ring) was 35.2 ± 7.11 pm/%, while that of MRR2 (the second ring) sensor was 98.3 ± 8.19 pm/%. In 2009, Fu [4] presented a long cycle sugar water sensor fabricated using a 410-µm long raster of a single-mode fiber, SMF28. In 2009, Binu presented another fiber sensor probe used for glucose measurements [5]. The laser wavelength of the fiber was 660 nm, the fiber length was 40 cm, the diameter was 1 mm, the numerical aperture was 0.5, the core refractive index was 1.492, and the cladding refractive index was 1.402. For glucose concentrations of 0–25%, its sensitivity was 0.0072 V/%, and the results indicated that the voltage was shifted by up to 1000 µm, with a peak value of up to 182 mv. In 2010, Yasin presented another fiber glucose concentration sensor [6]. The incident wavelength was 594 nm (using a helium neon yellow laser), the fiber length was 2 m, the diameter was 0.25 mm, the numerical aperture was 0.5, the core refractive index was 1.492, and the cladding refractive index was 1.402. The glucose concentration of 50 mL of deionized water was raised, respectively, by the addition of 2.5, 7.5, 10, or 12.5 g of glucose, and the peak sensitivity of the sensor was 0.0103 Mv/% with a linear regression value of 0.891, while its voltage shift sensitivity was 0.0229 mm/% with a linear regression value of 0.939.

In 2012, Srivastava [7] made a U-shaped plasma resonance fiber blood sugar sensor, where the surface of the fiber optic sensor was coated with metal nanometer particles with local surface plasma resonance features. Forming a U-shaped fiber not only enhanced the sensor sensitivity but also allowed for point-to-point measurement. Moreover, the surface quality of the fiber was changed by combining glucose oxidase with a 1% amino silicone halothane ethanol and acetic acid mixture. Next, gold nanoparticles were applied, and the sensor was soaked in a cystamine dihydrochloride solution that produced NH2, which allowed the glucose oxidase to adhere to it. When the glucose concentration ranged from 0 mg/dL to 250 mg/dL, the absorbance gradually declined as the concentration was increased. Laboratory results showed that, when the bend radius was 0.982 mm, the sensor achieved its maximum sensing sensitivity, a quicker response time, and the solution used only needed to be about 150 μL in volume. In 2014, Fallauto [8] fabricated a fiber sensor with a surface coated with a layer of gold as the surface of the plasma wave (SPR) for glucose concentration measurements. After the cladding of the fiber was removed, plasma sputter deposition on the fiber surface was conducted with 50 nm gold ions, halogen lamps, and a micro pump, after which the sensor was used for measurements of glucose concentrations ranging from 0 to 600 mg/dL. The lab results indicated that the sensitivity of the sensor was 1154 nm/RIU.

In 2015, Li [9] presented a U-shaped fiber attenuation total internal reflection (ATR) sensor implanted with silver nanoparticles that was used for glucose monitoring. The fiber was formed into a U-bend radius of 2.5 mm to increase its sensitivity, and it was coated with a biocompatible translucent film that could filter many biological elements in interstitial fluid while still allowing glucose molecules to pass through it. When the laser wavelength was 1081 cm^−1^, the glucose concentration sensitivity of the silver nanoparticle sensor was 0.00006444 a.u./(mg/dL), the linear regression was 0.986, and the resolution was 15 mg/dL. Without the silver nanoparticles painted onto it, the sensitivity of the sensor was only 0.00002172 a.u./(mg/dL), the linear regression was 0.989, and the resolution was 45 mg/dL. These results showed that the resolution of the silver nanoparticle-enhanced U-fiber sensor was three times higher than that of a traditional ATR sensor. In 2016, Yin used long cycle fiber grids and a microfluidic chip manufacturing process to make an ultra-sensitive fiber optic sensor [10], using multiple layers to enhance the signal and measure glucose oxidase (GOD). Hassan et al. [11] investigated in vitro sensing of glucose using a newly developed efficient optical fiber glucose sensor based on a compound parabolic concentrator (CPC)-tipped polymer optical fiber (POF). In 2017, Yuan [12] used glucose oxidase substrate material as well as the resonance on a plasma surface (surface plasmon resonance, SPR) to measure glucose.

Based on the above references, we determined that it is feasible to measure glucose with an optical fiber sensor, while the use of gold nanoparticles and glucose oxidase coating could significantly improve such optical fiber sensors in terms of the resolution of their measurements glucose solutions and compared with the sensors described in the literature, the sensor presented in this study is smaller and its sensitivity is higher. Therefore, the U-shaped optical fiber probe sensor proposed in this study was wet-etched to increase its sensitivity, fabricated using a flame heating process, and packaged in glass. Finally, glucose solutions were measured using the proposed U-shaped optical fiber probe sensor coated with gold nanoparticles and glucose oxidase, and its wavelength and transmission loss were then analyzed.

## 2. Working Principle of the U-Shaped Optical Fiber Probe

**Figure 1:**
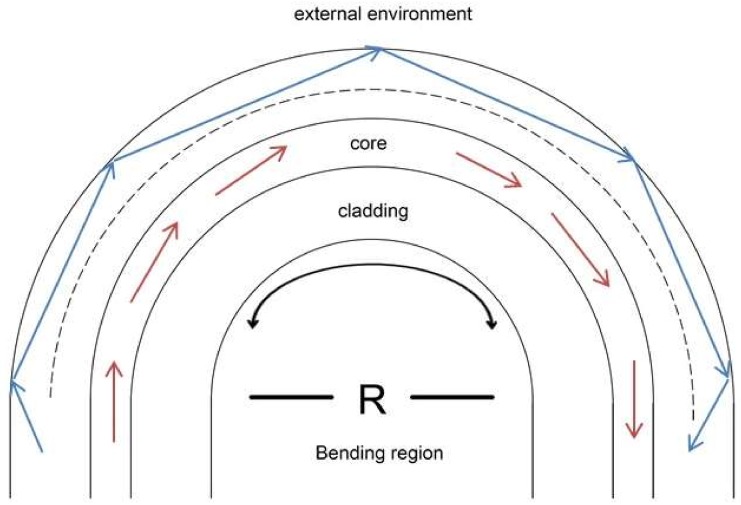
Schematic diagram of U-shaped optical fiber probe [13].

When the bending radius is as small as one degree, the phenomenon of mode interference in the spectrum can be observed. When the light source is incident to the curved part of the optical fiber, a leakage mode is generated because the fiber core layer and the cladding layer interface of the bent part no longer maintain total reflection, and part of the light source is excited from the fiber core layer to the cladding layer through the leakage mode. The light then reflects at the interface between the cladding layer and the external medium and then couples back to the fiber core layer to form a WGM mode, as shown in Figure 1.

In the U-shaped optical fiber sensor interference modal theory, the modal interference spectrum light source intensity is described as follows [13]:

(1) $$I=I_{co}+I_{wis}+2\sqrt{I_{co}+I_{wis}}\;\mathrm{COS}\left(\Phi \right)$$

where $I_{co}\;\mathrm{and}$ $I_{wis}$ are the light energy intensity of the core mode and the whispering gallery mode, respectively, and Φ is the phase difference between the cladding mode and the whispering gallery mode. The phase difference is described as follows [11]:

(2) $$\Phi =2\pi \left[n_{eff}^{co}\left(\lambda \right)-n_{clad}^{cl,m}\left(\lambda ,n_{ext}\right)\right]\frac{R}{\lambda_{D}}=\;\frac{2\pi \Delta n_{eff}R}{\lambda_{D}}\;=\left(2k+1\right)\pi$$

where $n_{eff}^{co}$ and $n_{clad}^{cl,m}$ are the effective refractive index core base mode and m level cladding mode, respectively, $\Delta n_{eff}$ is the effective refractive index difference between the core base mode and the m level cladding mode and can be described as $n_{eff}^{co}\left(\lambda \right)-n_{clad}^{cl,m}\left(\lambda ,n_{ext}\right)$, *R* is the bending radius of the fiber, $\lambda_{D}$ is the wavelength loss point position, and k is a constant. The optical fiber diameter is 1.11 mm, $n_{eff}^{co}$ is 1.4682, and $\lambda_{D}$ is 1550 nm.

When we satisfy Φ=(2k+1)π, the formula can be calculated as follows [14]:

(3) $$\lambda_{D}\;=\;\frac{2\Delta n_{eff}R}{2k+1}$$

From the above formula, we can see that different refractive indexes will result in different effective refractive index differences between the core mode and cladding mode, and that the wavelength loss point position will change according to the different refractive indexes. The sensitivity of the wavelength can be described as follows [11]:

(4) $$\frac{d\lambda_{D}}{dn_{ext}}\;=\;\frac{-\lambda_{D}}{\Delta n_{eff}}\frac{\partial n_{eff}^{cl,m}}{\partial n_{ext}}\;/\;\left[1-\frac{\lambda_{D}}{\Delta n_{eff}}\left(\frac{\partial n_{eff}^{co}}{\partial \lambda }-\frac{\partial n_{eff}^{cl,m}}{\partial \lambda }\right)\right]$$

where $\lambda_{D}$ is the loss point of the wavelength in Equation (4). When the refractive index is varied, the effective refractive index of the core mode and cladding mode will change accordingly, which will make the wavelength produce a displacement action. As such, we can detect different concentrations due to the wavelength shift caused by this phenomenon.

## 3. Experiment and Processing

### 3.1. Processing and Fabrication of U-Shaped Optical Fiber Probe

In this study, a single-mode fiber (SMF-28) was subjected to a flame heating method to form the fiber into the proposed U-shaped optical fiber sensor. When fibers with different diameters and etched radiuses are used, the resonance wavelength position can be controlled and the sensitivity of the fiber to changes in the external environment can be improved. For the etching process, a wire stripper was used to strip away about 4.5 cm of the intermediate protective layer of the fiber, and the exposed fiber was cleaned using alcohol, glued to the etching plate, and etched with a silicon dioxide solution (i.e., buffer oxide etch, BOE).

**Figure 2:**
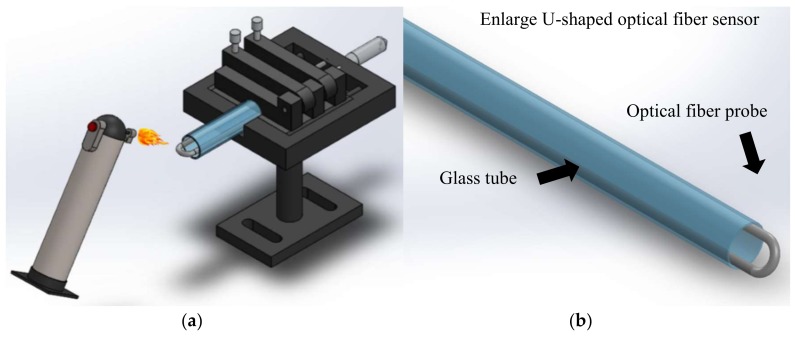
U-shaped optical fiber probe fabricated by flame heating. (**a**) the process of the U-shaped optical fiber probe (**b**) a magnified illustration of the U-shaped optical fiber probe.

The flame heating method was used after the etching of the fiber (to a diameter of 50 um) was finished. More specifically, the fiber was affixed to a micro platform, with a pre-tension stress applied on the fiber, and then a gas torch was used to heat the bend at the top of fiber. After this heat treating, the fiber was narrowed, as shown in Figure 2. As the glass transition temperature is reached (melting point 800 °C–1000 °C), the fiber structure will instantly become loose, allowing it to retract into the glass tube. After cooling, the U-shaped optical fiber probe can be obtained. To match that the position of the loss generate and the strongest signal strength position 1550 nm. The diameter must be designed around 1.11 mm.

**Figure 3:**
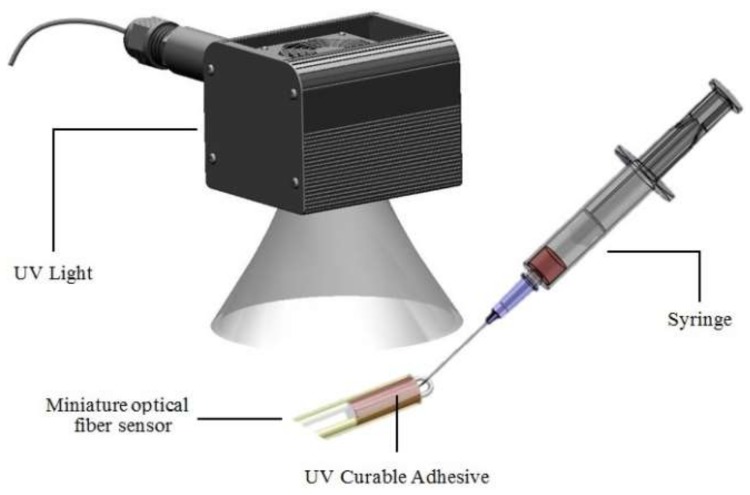
The sensor packaged by UV glue.

**Figure 4:**
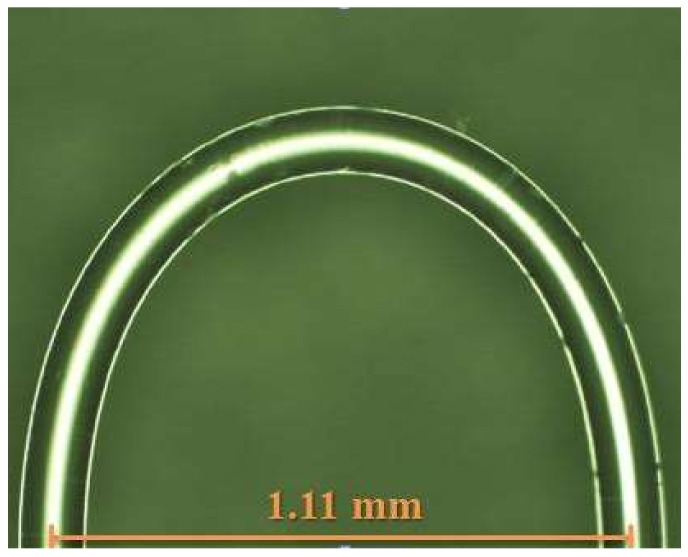
The sensor bend diameter.

After finishing the U-shaped optical fiber probe sensor, it was then packaged in a glass tube using UV glue to reduce its environmental impact and the effects of human factors. The glass tube was subjected to ultra-violet irradiation. Because this sensor is quite sensitive to changes in diameter, we needed it to be packaged to ensure that its diameter remained fixed. Figure 3 is a diagram of the U-shaped optical fiber probe package. Figure 4 is an optical microscope photograph of the U-shaped optical fiber probe showing the bend diameter.

### 3.2. Glucose Oxidase Coating and the Setup of the Glucose Sensing Experiment

First, the sensor was dipped in (3-aminopropyl) triethoxy silane (APTES, 97%) for 2 h to cause the silylation of the surface and then dipped in a solution containing gold nanoparticles (nanoComposix products, nanospheres was 100 nm) for 24 h to allow the gold nanoparticles to adhere to the fiber via electrostatic self-assembly. The U-shaped sensor was then rinsed in absolute ethanol for 5 min and DI water for 5 min. A 1 μM glucose oxidase (GOD) solution was then prepared in DI water and immobilized onto the gold nanoparticles that had adhered on the fiber surface for about 1 h. Finally, the U-shaped sensor was dipped in 1 mM phosphate buffered saline (PBS) solution for 10 min and then washed with DI water for 5 min to remove any unbound GOD.

**Figure 5:**
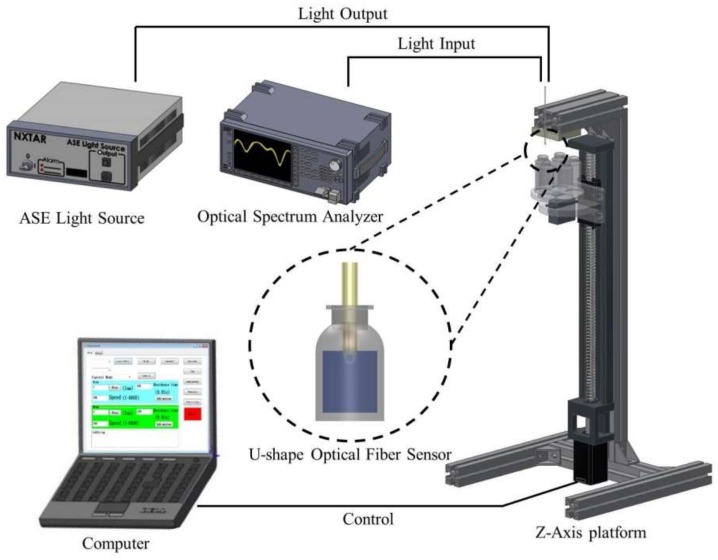
Experimental setup for glucose sensing.

The U-shaped optical fiber sensing glucose experimental set-up is shown in Figure 5. The amplified spontaneous emission (ASE-2200, ASE light source, NXTAR Technologies Inc., Tainan, Taiwan) light source was attached to one end of the sensor, while an optical spectrum analyzer (OSA) was attached to the other. A computer-controlled Z-axis platform was used to raise and lower the specimen so that we did not have to touch the sensor, thus reducing the potential for human error.

## 4. Results and Discussion

### 4.1. Non-Coating Glucose Sensing

**Figure 6:**
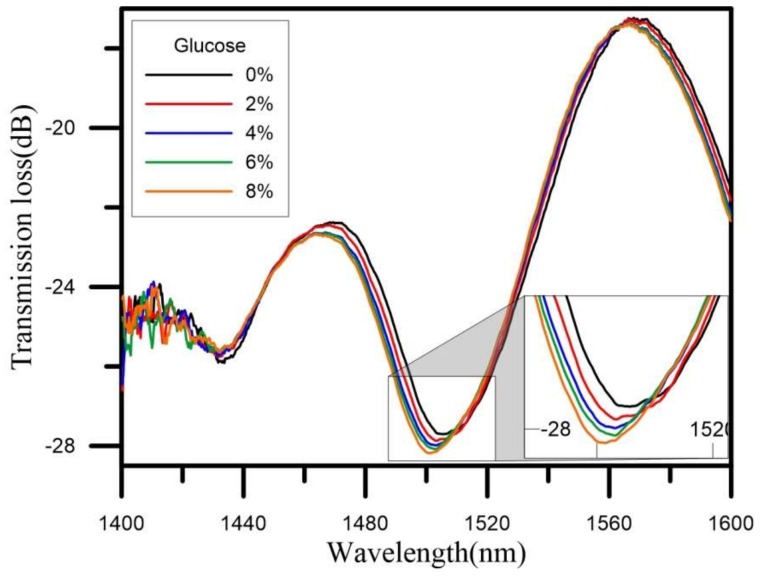
Glucose solution sensing spectrum.

In this study, the U-shaped optical fiber probe sensor was used to measure glucose solutions. To compare the effect of the sensing layer in the measurement, a version of the U-shaped optical fiber probe sensor without the sensing layer was first used to perform measurements. The glucose concentration ranged 0–8%, with the concentration being increased from low concentrations to high concentrations during this experiment. The results indicated that the refractive index became higher as the concentration was increased. As the glucose concentration was changed from 0% to 8%, the initial wavelength of 1506.25 nm was gradually shifted to shorter and shorter wavelengths, finally reaching 1502 nm, while the transmission loss was gradually increased from the initial −27.71 dB value to −28.10 dB. Figure 6 shows the U-shaped optical fiber probe measurements of the glucose solution sensing spectrum.

**Figure 7:**
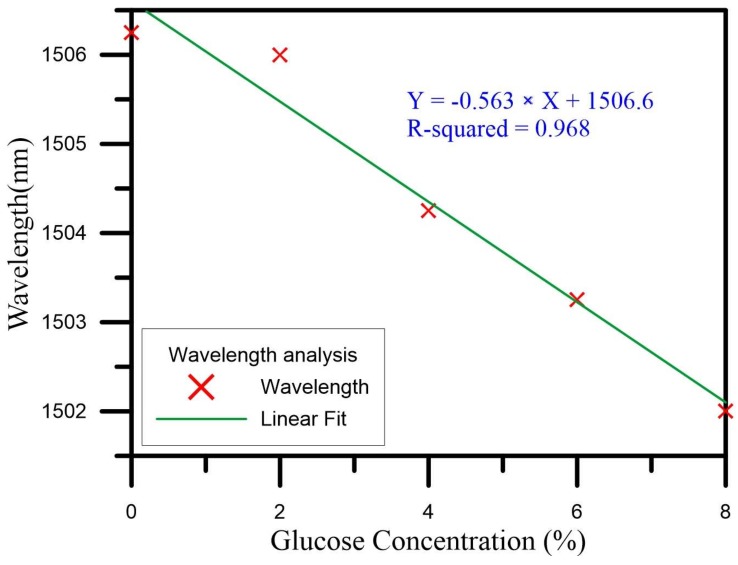
The analysis of wavelength.

**Figure 8:**
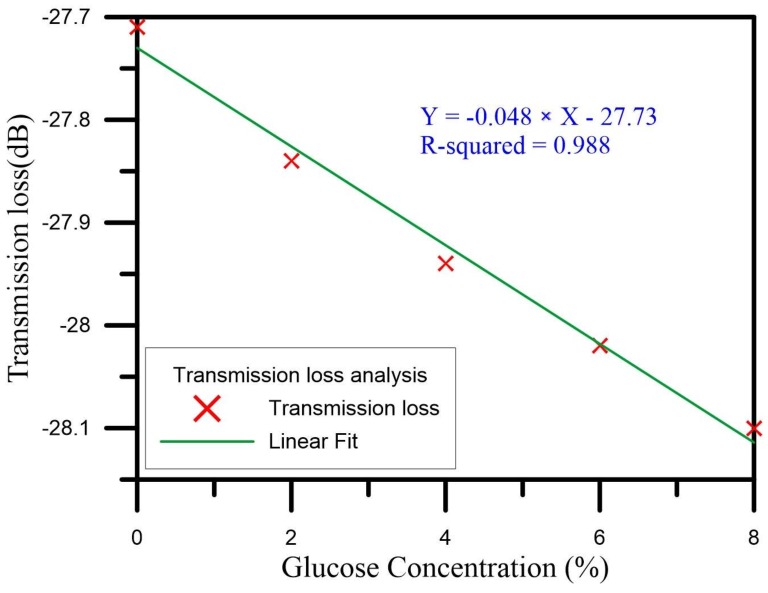
The analysis of transmission loss.

We then analyzed the wavelength and the transmission loss drawn with a graph on the spectrogram. For the wavelength drift for concentrations increasing from 0% to 8%, the initial wavelength of 1506.25 nm was gradually shifted to shorter and shorter wavelengths, finally reaching 1502 nm. The total amount of the drift was thus 4.25 nm, indicating a wavelength drift sensitivity of −0.563 nm/% and a linear regression value of up to 0.968. Figure 7 is a graph of the wavelengths of the U-shaped optical fiber probe sensor as it measured the glucose solutions of different concentrations. As for the transmission loss when the concentration was raised from 0% to 8%, the initial transmission loss of −27.71 dB was gradually increased via energy loss, finally reaching −28.10 dB. The change of the total loss was thus −0.39 dB, the loss sensitivity was −0.048 dB/%, and the linear regression value was up to 0.988. Figure 8 is a graph of the transmission loss values of the U-shaped optical fiber probe sensor as it measured the glucose solutions of different concentrations.

### 4.2. Glucose Sensing with the Gold Nanoparticle and Glucose Oxidase Coating

In the second phase, we used the U-shaped optical fiber probe sensor coated with gold nanoparticles and glucose oxidase for the glucose measurements, with the glucose concentration ranging from 0.1% to 0.5%. During the experiment, the concentration was raised from low concentrations to the high concentrations, and to test the sensing layer effects and reproducibility, we completed five cycles of the experiment. The following analysis discusses the wavelength and transmission loss of the sensor for the five cycles of the experiment.

**Figure 9:**
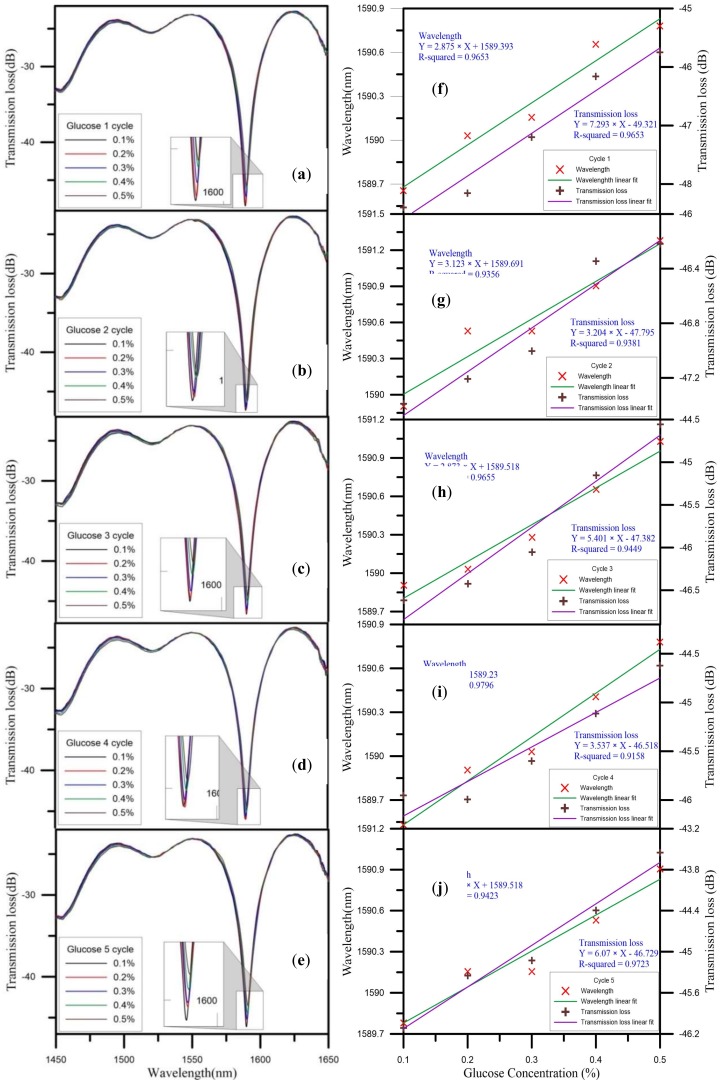
Cycle 1–5 glucose solution sensing spectra (**a**–**e**); and analysis of wavelength and transmission loss (**f**–**j**).

**Figure 10:**
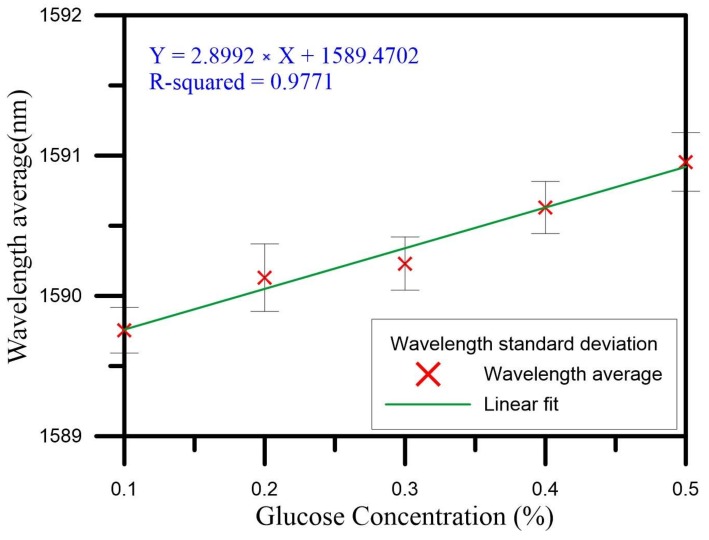
The standard deviation analysis of wavelength of Cycles 1–5.

**Figure 11:**
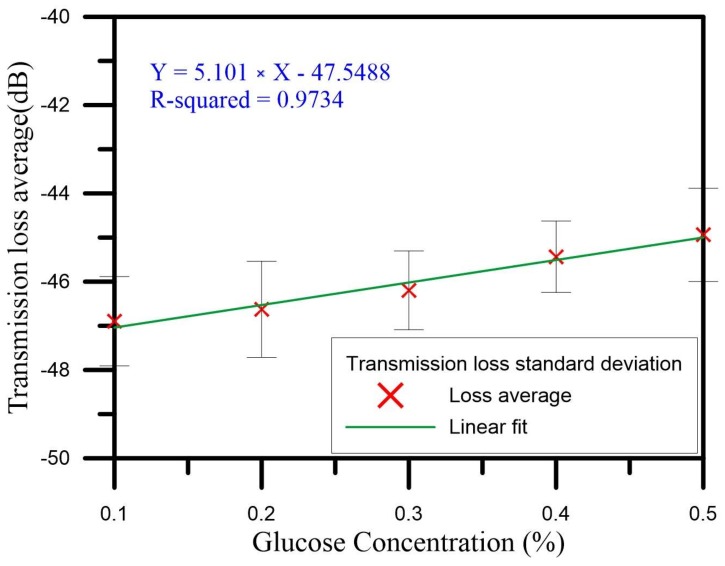
The standard deviation analysis of transmission loss for Cycles 1–5.

In Cycles 1–5, the concentration changed from 0.1% to 0.5%. The initial wavelength gradually drifted to longer wavelengths. In the measurements from all five cycles, the highest wavelength drift sensitivity was 3.123 nm/%, which is shown in Figure 9g, and the linear regression value was 0.9356. The initial wavelength of 1589.905 nm drifted to 1591.279 nm, so the total amount of drift was 1.374 nm. In the measurements from all five cycles, the highest transmission loss sensitivity was 7.293 dB/%, which is shown in Figure 9f, and the linear regression value was 0.9652. The initial transmission loss of −48.399 dB was reduced to −45.749 dB, so the total loss variation was 2.56 dB. Figure 9 depicts the glucose solution sensing spectrum for Cycles 1–5 and the analysis of the wavelength and transmission loss. We averaged the measurements for the five cycles in analyzing the wavelength and transmission loss. The average wavelength drift sensitivity was 2.899 nm/%, and the liner regression value was 0.9771. Figure 10 shows the standard deviation analysis results for the wavelength during Cycles 1–5. The average transmission loss was 5.101 dB/%, and the linear regression value was 0.9734. Figure 11 shows the standard deviation analysis results for the transmission loss during Cycles 1–5.

From the above experimental results, we know that the concentrations measured by the U-shaped optical fiber probe sensor without the gold nanoparticle and glucose oxidase coating ranged 0–8%. The wavelength drift sensitivity was −0.563 nm/%, and the linear regression value was 0.968. In addition, the loss sensitivity was −0.048 dB/%, and the linear regression value was 0.988. These results showed that the uncoated U-shaped optical fiber probe sensor provided good glucose measurements, while the U-shaped optical fiber probe sensor coated with gold nanoparticles and glucose oxidase was used to measure glucose concentrations of 0.1–0.5%. In Cycles 1–5 of measurement, the highest wavelength drift sensitivity was 3.123 nm/%, and the linear regression value was 0.9356. In Cycles 1–5 of measurement, the highest loss sensitivity was 7.293 dB/%, and the linear regression value was 0.9652. Compared with the U-shaped optical fiber probe sensor without the gold nanoparticle and glucose oxidase coating, the wavelength drift sensitivity was 5.547 times higher and the loss sensitivity was 151.94 times higher. This shows that the U-shaped optical fiber probe sensor coated with gold nanoparticles and glucose oxidase had a significant improvement in terms of the sensitivity of its glucose solution measurements, which would also be significantly helpful for the measurement of glucose solutions with lower concentrations. The size of the sensor is also much smaller than the sizes of the sensors discussed in the previous literature, which allows it to save space and to improve the sensitivity. It was also shown by the five cycles of the experiment that the reproducibility of the U-shaped optical fiber probe sensor coated with the sensing layer is good.

## 5. Conclusions

In this study, we successfully applied a coating of gold nanoparticles and glucose oxidase on a U-shaped optical fiber probe sensor and then used the sensor to measure concentrations of from 0.1% to 0.5% glucose solution. The wavelength drift sensitivity of the sensor reached up to 3.123 nm/%, and the linear regression value was 0.9356. In addition, the transmission loss sensitivity reached up to 7.293 dB/%, and the linear regression value was 0.9652. The wavelength drift sensitivity was thus 31.87 times higher than the 98.3 ± 8.19 pm/% value reported for the sensor presented in one of the above-mentioned reference studies [1]. The radius of the sensor was also 0.555 mm better than the 0.982 mm [5] and 2.5 mm [7] values reported in previous studies. The resolution translated into a value of 0.16 mg/dL, which was 93.75 times higher than the 15 mg/dL value reported in a previous study [7]. This shows that the U-shaped optical fiber probe sensor has a very high research and development value, and, in the future, the goal will be to use the sensor for human blood glucose measurements for biomedical purposes.
